# Hiatal Hernia of Stomach and Lesser Omentum in a Cadaver: Is It a Type III or IV?

**DOI:** 10.7759/cureus.55431

**Published:** 2024-03-03

**Authors:** Luis A Alvarez, Alyssa M Debski, Anna E Egli, Morgan A Hatlovic, Oren D Rosenthal, Seth Gardner

**Affiliations:** 1 Department of Anatomy, Lake Erie College of Osteopathic Medicine Bradenton, Bradenton, USA

**Keywords:** hiatal hernia classification, lesser omentum, giant hiatal hernia, type iv, paraesophageal, aneurysm, hiatal hernia

## Abstract

Hiatal hernias, protrusions of abdominal viscera through the esophageal hiatus, are classified into four types. Types I and II involve ascent of the stomach without affecting the gastroesophageal junction. Types III and IV involve the gastroesophageal junction. Type IV specifically may have stomach as well as other abdominal organ involvement, such as pancreas or omentum. Among these types, type IV is the most complex and rare form, accounting for only 0.1% of all cases of hiatal hernias. This report presents a case of a type IV hiatal hernia involving the lesser omentum and a significant portion of the stomach in an 86-year-old male cadaver with a history of mediastinal surgery. To our knowledge, this presentation in a cadaver has not previously been reported in the literature.

This case highlights classification inconsistencies in the literature, particularly regarding type IV hiatal hernias. It is unclear given the current classification system, whether this presentation would be considered a type III or type IV hiatal hernia as it fits both criteria and there are several interpretations of the criteria of a type IV hiatal hernia. Inconsistencies in the classification system may impede standardization of care. This report highlights the need for a more precise classification system that better accounts for anatomical changes and clinical presentation.

## Introduction

Hiatal hernias are structural abnormalities in which abdominal viscera protrude through the esophageal hiatus of the diaphragm [1]. According to Kim et al., the first classification system in 1926 separated hiatal hernias into three distinct types as follows: type I, type II, and type III [2]. In 1954, Barrett updated the hiatal hernia classification system to include a type IV hiatal hernia, which is still in use today [1]. Kim et al. argue that improvements to the classification system are necessary after 70 years of use [2].

Four types of hiatal hernias are differentiated based on anatomical involvement and mechanism of herniation [2]. Type I involves a symmetrical ascent of the stomach through the diaphragmatic crura [1]. Type II is the displacement of a portion of the stomach into the mediastinum through the esophageal hiatus of the diaphragm without affecting the gastroesophageal junction [3]. Type III is similar to type II but involves protrusion of the gastric fundus into the mediastinum, including the gastroesophageal junction [3]. Type IV hernias, sometimes referred to as giant hiatal hernias, involve herniation of the stomach as well as the omentum or other abdominal organs into the mediastinum. Herniation of the omentum, bowel, spleen, or pancreas does not change the type IV classification of the hernia [2,4-6].

The etiology of hiatal hernias includes the widening of the esophageal hiatus from acquired or congenital means, increased abdominal pressure, and/or esophageal shortening [7]. One risk factor for these includes increased intra-abdominal fat associated with obesity. The intra-abdominal fat predisposes gastric contents toward migrating superiorly in the digestive tract as well as separation of the diaphragmatic crura or lower esophageal sphincter due to excess stretch or pressure, ultimately leading to esophageal injury or gastroesophageal reflux disease (GERD) [7].

The symptoms associated with hiatal hernias occur because they disrupt the normal functioning of the lower esophageal sphincter and the diaphragmatic crura [6]. This disruption can result in either the backflow of stomach contents into the esophagus (reflux) or the retention of gastric contents within the hernia itself [6]. The most common type of hiatal hernias, type I, are typically asymptomatic or present with symptoms of GERD and are treated by usual management strategies for GERD [3]. Both type II and III hiatal hernias are paraesophageal and may present with epigastric pain, chest pain, substernal fullness, shortness of breath, nausea, or vomiting [7]. Type IV hiatal hernia clinical presentation is most variable, ranging from asymptomatic cases to cases involving organ strangulation. Notably, Goldman and Andrew define large hiatal hernias as having at least one-third of the stomach in the chest cavity, rather than multiple organ involvement [7]. We report a type IV hiatal hernia presentation and explore its potential etiology. Additionally, we highlight inconsistencies in hiatal hernia classification.

## Case presentation

A routine anatomy dissection of an 86-year-old male donor cadaver, preserved in formalin, was completed at Lake Erie College of Osteopathic Medicine in Bradenton. The cadaver was donated to the medical school without any personal identifiers. The listed causes of death include aspiration pneumonitis, oropharyngeal dysphagia, probable end-stage vascular dementia with behavioral disturbance, and cerebral atherosclerosis. Other significant comorbidities included anorexia, weight loss, hypertension, hyperlipidemia, cardiomyopathy, unspecified heart failure, mitral valve insufficiency, atrial fibrillation, Chronic Obstructive Pulmonary Disease (COPD), depression, and alcohol dependence in remission. The cadaver had multiple sternotomy wires on the anterior chest wall from mediastinal surgery.

**Figure 1:**
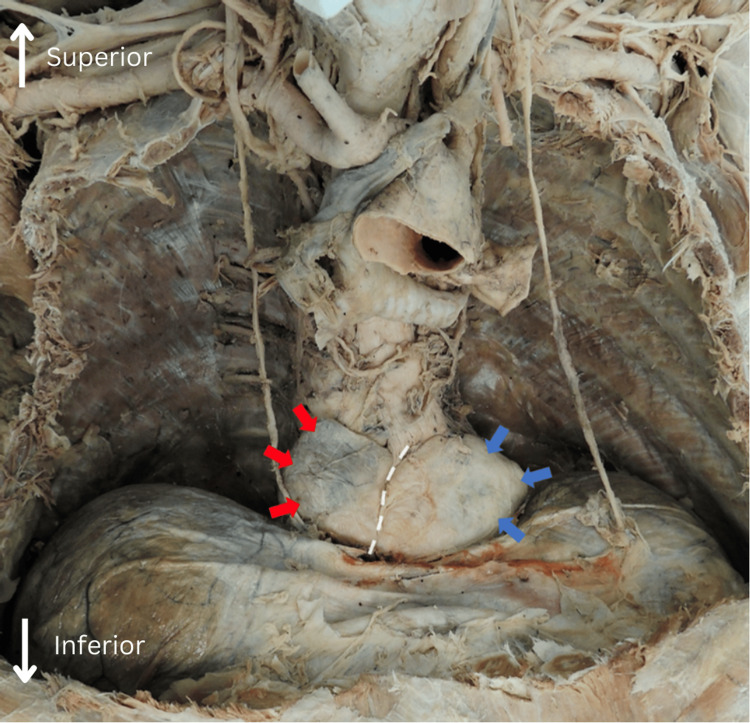
Anterior to posterior view of the thoracic cavity with the heart and lungs resected. The dashed line indicates the delineation between the tissue masses. Red arrows indicate lesser omentum. Blue arrows indicate the lateral margin of the gastric fundus.

During the removal of the anterior thoracic wall, a hiatal hernia was identified. Examination revealed involvement of the fundus and cardia of the stomach (Figure 1, blue arrows) and the lesser omentum (Figure 1, red arrows). This indicates a type IV hiatal hernia, characterized by the herniation of structures in addition to the stomach, in this case, the lesser omentum.

**Figure 2:**
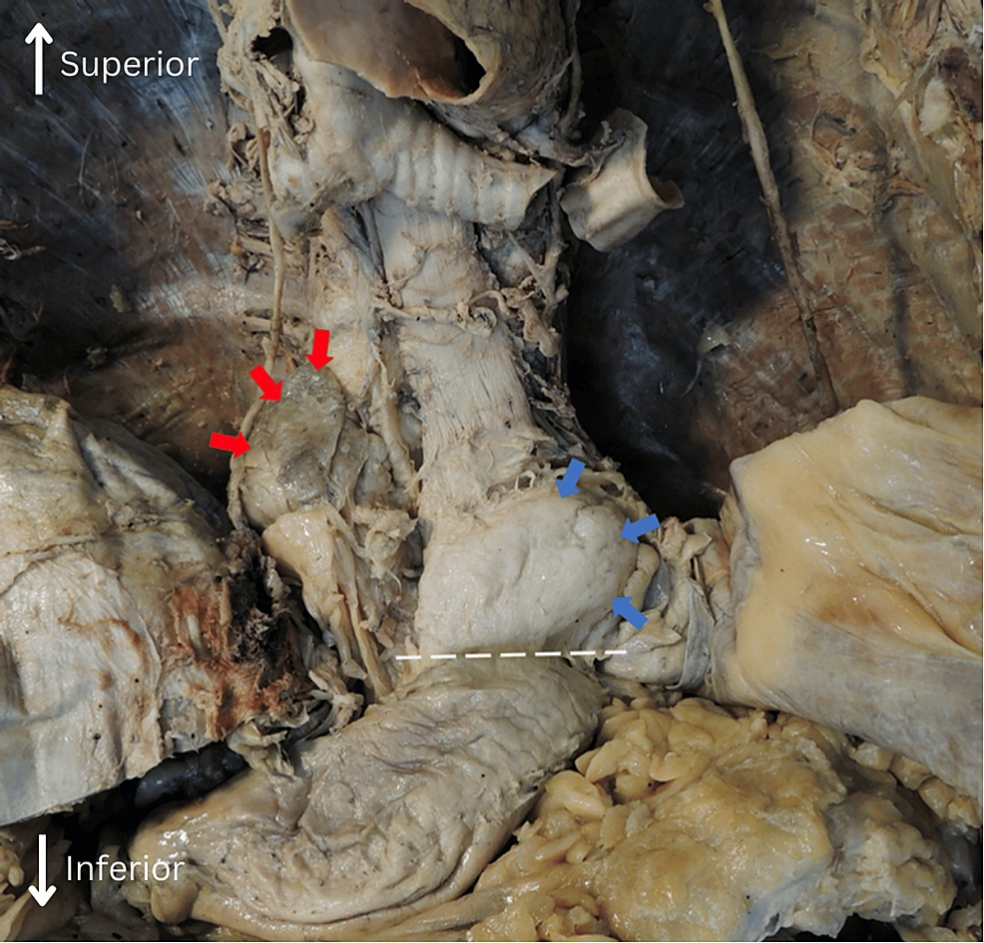
Anterior to posterior view of the thoracic cavity. A portion of the diaphragm is resected to further visualize the hiatal hernia. The red arrows indicate dissected margins of the lesser omentum. The blue arrows indicate the lateral margins of the gastric fundus. The white dashed line demonstrates where the esophageal hiatus resided.

The diaphragm was dissected free from around the masses, further revealing the hiatal hernia composed of the lesser omentum (Figure 2, red arrows) and approximately one-third of the stomach (Figure 2, blue arrows). The left gastric artery was displaced superiorly with the herniation. Figure 2 demonstrates white dashed lines that demarcate the level of the esophageal hiatus, allowing for visualization of the constriction of the stomach (Figure 2, white dashed line). Notably, a 2 cm abdominal aortic aneurysm was also discovered upon dissection of the abdominal cavity.

## Discussion

Type IV hiatal hernias are rare, representing only 0.1% of all hiatal hernia cases [8]. They are typically defined as the stomach along with the omentum or other abdominal organs, bowel, pancreas, or spleen herniating into the mediastinum [6]. This case involves the lesser omentum, the gastric cardia, and fundus herniating into the thoracic cavity, designating it as a type IV hiatal hernia according to the currently accepted classification system [3]. We found a report of the greater omentum herniating with the stomach but did not find any reports that included descriptions of the lesser omentum [9].

The etiology of hiatal hernias remains unclear due to a high prevalence of asymptomatic cases that may only be incidentally detected [10]. Currently, there are three major hypotheses on hiatal hernia pathogenesis; widening of the esophageal hiatus from acquired or congenital means, increased abdominal pressure, and although more controversial, esophageal shortening [11]. Mitiek and Andrade reported GERD and subsequent esophageal shortening specifically with type IV hiatal hernia formation [12]. In the present case, there was no mention of GERD in the limited history provided. In this cadaver, sternotomy wires on the anterior chest wall indicate past mediastinal surgery. Mediastinal surgery may disrupt the esophageal hiatus leading to greater laxity [11]. Gastric dilatation has been reported to occur postoperatively in cardiothoracic surgeries and may influence the development of hiatal hernias as well [13].

Impingement of the lesser omentum may lead to vascular consequences. The left gastric artery, for example, travels into the lesser omentum adjacent to the lesser curvature of the stomach [14]. The hepatoduodenal ligament, a subcomponent of the lesser omentum, carries the portal triad. The present case demonstrated significant displacement of the left gastric artery as it followed the lesser omentum superiorly. Additionally, reports of small bowel loops and the greater omentum herniating through the lesser omentum via the foramen of Winslow or through anatomical defects are documented in the literature [9,15]. In these cases, the main concern is strangulation of the small bowel or greater omentum, not the lesser omentum. In the present case, the left gastric artery and the hepatoduodenal ligament did not herniate into the posterior mediastinum with the rest of the lesser omentum or the stomach, suggesting that clinically relevant ischemia of the stomach or lesser omentum is unlikely.

The classification of hiatal hernias in the literature is inconsistent [16-18]. For example, Ceron et al. in 2022 found that only one clinical case out of 846 searches met the criteria of a classic type II hiatal hernia, despite labeling each case as a type II hiatal hernia [16]. Similarly, varying criteria for type IV hiatal hernias have been published. Some sources state that as long as there is more than one-third to one-half of the stomach through the esophageal hiatus, it is considered a type IV hiatal hernia, regardless of the involvement of other organs [7,17,18]. Others define a type IV hiatal hernia as the herniation of abdominal organs as well as the stomach into the mediastinum through the esophageal hiatus [2,4-6]. The case presented here most closely satisfies the latter definition of a type IV hiatal hernia; the lesser omentum and stomach both herniating into the mediastinum. It is possible that the latter definition is merely an evolution of type III hiatal hernias, which, as discussed previously, involves only the gastric fundus and the gastroesophageal junction protruding into the mediastinum [6,17]. The current classification system does not account for the variability among hiatal hernia symptom presentations and has less value in clinical decision-making [2]. 

Type IV hiatal hernias are unique because their clinical consequences can be more extensive depending on the organs involved. Like type I-III hiatal hernias, type IV hiatal hernias are associated with gastric reflux, dysphagia, early satiety, anemia, and pain due to the displacement of the esophageal junction and compression of the stomach within the hiatus [18,12]. Furthermore, additional organ strangulation distinctively becomes the main concern for surgeons when identifying a type IV hiatal hernia [19]. Herniation of the small bowel, spleen, and pancreas has been reported in cases of type IV hiatal hernias. Subsequent compression and strangulation of these organs can be devastating, leading to organ insufficiency or even rupture [19].

Nevertheless, even asymptomatic cases of major organ involvement, such as the pancreas, have also been reported [20]. Due to the highly variable clinical presentation, clinicians face the challenge of identifying the necessity for surgical intervention versus monitoring asymptomatic patients with the potential of acute situations eventually arising [2]. To improve the classification system, we suggest type IV hiatal hernias can be subdivided depending on which specific abdominal organs herniate as well as the specific clinical presentation of the patient. This incongruity in the classification system may provide an explanation for the scarcity of research pertaining to type IV hiatal hernias that involve the lesser omentum. It is possible that instances of the lesser omentum and stomach protruding into the posterior mediastinum are more common, yet categorizing these occurrences as type IV hiatal hernias fails to encompass the vast variability in the clinical presentation of each individual case. Accurately classifying hiatal hernias by considering both anatomy and clinical presentation can help standardize patient care.

There are varying opinions regarding the standard treatment for paraesophageal hiatal hernias [12,18]. Generally, the decision to undergo surgical intervention is based on symptoms [12]. Symptomatic patients undergo surgery with the main goal being to reduce the herniated sac and organs involved [10,12]. Laparoscopic or transthoracic open surgery can be performed, depending on the complexity of the hernia [12]. If patients suffer from GERD, then fundoplication is typically performed as well [12]. Managing asymptomatic patients presents a challenge to clinicians. Clinicians debate between managing asymptomatic patients conservatively or with surgical repair to prevent future complications [18]. Conservative management involves observation and symptomatic relief with medication such as proton pump inhibitors [12]. It has been found, though, that without surgical intervention, symptoms progress in up to 45% of patients with giant hiatal hernias, emphasizing the importance of accurate classification [14]. Surgeons must decide whether preventing symptomatic complications with prophylactic surgery outweighs the potential complications associated with surgery.

## Conclusions

To our knowledge, there are no reports in the literature that describe a cadaveric case of a type IV hiatal hernia involving the lesser omentum. This report presents a novel type IV hiatal hernia presentation demonstrating approximately one-third of the stomach with fundus and lesser omentum protruding through the esophageal hiatus. We emphasize the need for enhancing the current hiatal hernia classification system through this case’s novelty. Creating a more precise classification system will help standardize patient care. The development of a type IV hiatal hernia is likely multifactorial, with research suggesting that increased abdominal pressure, obesity, and esophageal shortening may play a role in development. Our report of a type IV hiatal hernia involving the lesser omentum offers insight into type IV hiatal hernia structure, classification, and pathogenesis.

## References

[1] Kim P, Turcotte J, Park A (2021). Hiatal hernia classification-way past its shelf life. Surgery.

[2] Barrett NR (1954). Hiatus hernia: a review of some controversial points. Br J Surg.

[3] Skinner DB (1985). Hernias (hiatal, traumatic, and congenital). Gastroenterology.

[4] Davis SS Jr (2008). Current controversies in paraesophageal hernia repair. Surg Clin North Am.

[5] Dunn CP, Patel TA, Bildzukewicz NA, Henning JR, Lipham JC (2020). Which hiatal hernias need to be fixed? Large, small or none?. Ann Laparosc Endosc Surg.

[6] Abbara S, Kalan MM, Lewicki AM (2003). Intrathoracic stomach revisited. AJR Am J Roentgenol.

[7] Goldman L, Andrew IS (2019). Diseases of the esophagus. Goldman-Cecil Medicine. Twenty-Sixth Edition.

[8] Krause W, Roberts J, Garcia-Montilla RJ (2016). Bowel in chest: type IV hiatal hernia. Clin Med Res.

[9] Tanaka Y, Saika Y, Asao Y, Tanaka M, Nohara R (2020). Omental herniation through the esophageal hiatus: a rare cause of gastric outlet obstruction and its CT findings. Radiol Case Rep.

[10] Oleynikov D, Jolley JM (2015). Paraesophageal hernia. Surg Clin North Am.

[11] Bittner R, Köckerling F, Fitzgibbons RJ Jr, LeBlanc KA, Mittal SA, Chowbey P (2018). Laparo-Endoscopic Hernia Surgery: Evidence Based Clinical Practice. Springer.

[12] Mitiek MO, Andrade RS (2010). Giant hiatal hernia. Ann Thorac Surg.

[13] Song KJ, Yip R, Chung M (2022). New or enlarging hiatal hernias after thoracic surgery for early lung cancer. JTCVS Open.

[14] Luketich JD, Raja S, Fernando HC (2000). Laparoscopic repair of giant paraesophageal hernia: 100 consecutive cases. Ann Surg.

[15] Alagoa João A, Aparício D, João P, Pignatelli N, Nunes V (2022). Spontaneous internal hernia through a defect in the hepatogastric ligament. Radiol Case Rep.

[16] Ceron RE, Yates RB, Wright AS, Rodriguez HA, Lopez RG, Pellegrini CA, Oelschlager BK (2023). Type II hiatal hernias: do they exist or are they actually parahiatal hernias?. Surg Endosc.

[17] Migaczewski M, Grzesiak-Kuik A, Pędziwiatr M, Budzyński A (2014). Laparoscopic treatment of type III and IV hiatal hernia - authors' experience. Wideochir Inne Tech Maloinwazyjne.

[18] Pierre AF, Luketich JD, Fernando HC, Christie NA, Buenaventura PO, Litle VR, Schauer PR (2002). Results of laparoscopic repair of giant paraesophageal hernias: 200 consecutive patients. Ann Thorac Surg.

[19] Baison GN, Aye RW (2021). Complex and acute paraesophageal hernias - type IV, strangulated, and irreducible. Ann Laparsc Surg.

[20] Katz M, Atar E, Herskovitz P (2002). Asymptomatic diaphragmatic hiatal herniation of the pancreas. J Comput Assist Tomogr.

